# Retraction: Mechanosensitive channel candidate MCA2 is involved in touch-induced root responses in *Arabidopsis*

**DOI:** 10.3389/fpls.2015.00153

**Published:** 2015-03-05

**Authors:** Frontiers in Plant Science Editorial Office

**Keywords:** mechanosensitive channel, Arabidopsis, root, skewing, waving, calcium, touch response, mechanical stress

The authors and the journal wish to retract the 21 Aug 2014 article cited above in light of new experimental evidence.

Following the publication of the study, we performed a DNA microarray analysis to detect genes with expression levels that were specifically lower in the *mca2*-null mutant than in the Col-0 wild type, and found that the expression level of the *AXR4* gene (At1G54990), which encodes a protein required for the subcellular localization of the auxin influx carrier AUX1 (Dharmasiri et al., 2006), was significantly lower in the *mca2*-null mutant. To confirm this finding, we then performed a semi-quantitative reverse transcription-PCR analysis using the primers axr4-f1 and axr4-Cr1 (Figure 1A), and found that the RT-PCR product was detectable in some *mca2*-null seedlings at wild-type levels but not in other *mca2*-null seedlings at all. This result suggested that some *mca2*-null seedlings have a certain lesion in the *AXR4* locus, and a PCR-based genomic deletion analysis (Figures 1B,C) followed by DNA sequencing confirmed this speculation. Our conclusion is that most of the *mca2*-null seedlings used in the study presented in the above paper had a homozygous 2592-bp deletion that started from the intron between exons 1 and 2 of the *AXR4* gene and reached the intron between exons 1 and 2 of the adjacent gene *AT1G55000* (Figure 1A). Therefore, the phenotypes presented in the above paper may be ascribable to the *axr4* mutation, the *at1g55000* mutation, or both or even triple mutations, but not to the *mca2*-null mutation. The *AT1G55000* gene encodes the peptidoglycan-binding LysM domain-containing protein involved in a macromolecule catabolic process in the cell wall^1^.

**Figure 1:**
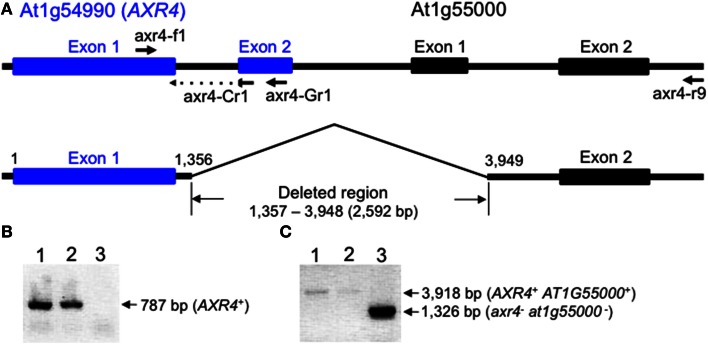
**Schematic representation of the *AXR4* locus and PCR data to check its integrity. (A)** The *AXR4* locus and its adjacent locus At1g55000 in *Arabidopsis thaliana* (ecotype Col-0). The upper line drawing shows intact loci, while the lower one shows loci with the 2592-bp deletion found in some *mca2*-null mutants. The position and orientation of the primers (axr4-f1, axr4-Cr1, axr4-Gr1, and axr4-r9) are presented by arrows. Nucleotide numbering starts from the 5′-end of exon 1 in the *AXR4* gene. **(B)** An example of PCR data with the primers axr4-f1 and axr4-Gr1. **(C)** An example of PCR data with the primers arx4-f1 and axr4-r9. Genomic DNA was isolated from an *mca2*-null mutant with the *mca2*^−^/*mca2*^−^ *AXR4^+^/AXR4^+^* genotype (lane 1), the wild type with the *MCA2^+^/MCA2^+^ AXR4^+^/AXR4^+^* genotype (lane 2), and another *mca2*-null mutant with the *mca2*^−^/*mca2^−^* *axr4^−^/axr4^−^* genotype (lane 3), and subjected to PCR followed by agarose gel electrophoresis. The nucleotide sequences of the primers axr4-f1, axr4-Cr1, axr4-Gr1, and axr4-r9 were 5′-GCAACGTGTAGCTAAGGCTCTTCC-3′, 5′-TCGCCGGATTTGCTTTCCTGAG-3′, 5′-GTAGTAGTCCATTCCTCACCAAG-3′, and 5′-AGCTCCATCTTCGTGTCTAG-3′, respectively, where the underlined nucleotide sequence is complementary to the coding strand of the 3′-terminal region of the exon 1 of the *AXR4* gene. The PCR conditions used were as follows: initial activation of Taq DNA polymerase (Quick Taq™ HS DyeMix, TOYOBO, Osaka, Japan) at 94°C for 2 min, followed by 30 cycles of denaturation at 94°C for 30 s, annealing at 55°C for 30 s, and extension at 68°C for 2 min.

Our phenotypic study revealed that none of the *mca2*-null *AXR4*^+^ *AT1G55000*^+^ seedlings showed all the abnormal phenotypes reported in the above paper, regarding the skewing, waving, and bending responses of the root. In contrast, the seedlings of the *axr4-1* (Ws-2 background) and *axr4-2* (Col background) single mutants obtained from the Arabidopsis Biological Resource Center (ABRC germplasm names CS8018 and CS8019, respectively) showed the same abnormal phenotypes as those described in the above paper. We also confirmed that the abnormal phenotypes for the skewing, waving, and bending responses of the *mca2*-null *arx4 at1g55000* triple mutant were identical to those of the *axr4-1* and *axr4-2* single mutants. These findings clearly demonstrated that the abnormal phenotypes described in the above paper were ascribed solely to the mutation in the *AXR4* gene.

An important question is why did some of our *mca2*-null germplasms have the *axr4*^−^*/axr4*^−^ *at1g55000*^−^*/at1g55000*^−^ allele? We had never used *axr4* mutants in our laboratory before the above paper was published. We speculated that some of the seeds of the *mca2*-null mutant (germplasm name: SALK_129208) obtained from the ABRC 12 years ago were heterozygous for the *AXR4* and *AT1G55000* loci (i.e., *AXR4^+^/axr4*^−^ *AT1G55000*^+^*/at1g55000*^−^), and multiple self-pollinations performed by us to maintain seed viability produced seed stocks with *axr4*^−^*/axr4*^−^ *at1g55000*^−^*/at1g55000*^−^ as well as *AXR4^+^/AXR4^+^ AT1G55000*^+^*/AT1G55000*^+^ and *AXR4^+^/axr4*^−^ *AT1G55000*^+^*/at1g55000*^−^, although all of the 20 seeds of the *mca2*-null mutant (germplasm name: SALK_129208C), which were newly obtained from the ABRC and tested, had the genotype of *AXR4^+^/AXR4^+^ AT1G55000*^+^*/AT1G55000*^+^. As for the *MCA2* locus, 19 out of the 20 seeds had the *mca2*^−^*/mca2*^−^ genotype and one had the *MCA2*^+^*/mca2*^−^ genotype. The *mca1*-null *mca2*-null double mutant and *mca2/MCA2* complementation lines used in the above paper were *AXR4^+^/AXR4^+^ AT1G55000*^+^*/AT1G55000*^+^. Furthermore, the *mca2*-null single and *mca1*-null *mca2*-null lines used in our previous study (Yamanaka et al., 2010) were also *AXR4^+^/AXR4^+^ AT1G55000*^+^*/AT1G55000*^+^.

We deeply regret any scientific misconceptions that have been caused by the above paper and apologize to the scientific community for any adverse consequences.

## Conflict of interest statement

The authors declare that the research was conducted in the absence of any commercial or financial relationships that could be construed as a potential conflict of interest.

## Footnotes

^1^Database of The Arabidopsis Information Resource (TAIR) at http://www.arabidopsis.org/servlets/TairObject?type=locus&name=AT1G55000

## References

[B1] Dharmasiri S. Swarup R. Mockaitis K. Dharmasiri N. Singh S. K. Kowalchyk M.. (2006). AXR4 is required for localization of the auxin influx facilitator AUX1. Science 312, 1218–1220. 10.1126/science.1122847 16690816

[B2] Yamanaka T. Nakagawa Y. Mori K. Nakano M. Imamura T. Kataoka H.. (2010). MCA1 and MCA2 that mediate Ca^2+^ uptake have distinct and overlapping roles in Arabidopsis. Plant Physiol. 152, 1284–1296. 10.1104/pp.109.147371 20097794 PMC2832256

